# Bioinformatic and Functional Analysis of a Key Determinant Underlying the Substrate Selectivity of the Al Transporter, Nrat1

**DOI:** 10.3389/fpls.2018.00606

**Published:** 2018-05-07

**Authors:** Muxue Lu, Guangzhe Yang, Peifang Li, Zhigang Wang, Shan Fu, Xiang Zhang, Xi Chen, Mingxing Shi, Zhenhua Ming, Jixing Xia

**Affiliations:** State Key Laboratory of Conservation and Utilization of Subtropical Agro-Bioresources, College of Life Science and Technology, Guangxi University, Nanning, China

**Keywords:** Nrat1, aluminum, Al transporter, selectivity, bioinformatic analysis

## Abstract

Nrat1 is a member of the natural resistance-associated macrophage protein (Nramp) family of metal ion transporters in all organisms. Different from other Nramp members capable of transporting divalent metals, Nrat1 specifically transports trivalent aluminum (Al) ion. However, molecular mechanism underlying the Al transport selectivity of Nrat1 remains unknown. Here, we performed structure-function analyses of Nrat1 and other Nramp members to gain insights into the determinants of ion selectivity. A phylogenetic analysis showed that plant Nramp transporters could be divided into five groups. OsNrat1 was found in one of the individual clades and clustered with SbNrat1 and ZmNrat1 on the evolutionary tree. Structural modeling revealed that Nrat1 transporters adopted a common LeuT fold shared by many Nramp-family transporters that likely employed an identical transport mechanism. Sequence alignment and evolutionary conservation analysis of amino acids identified a metal-permeation pathway of Nrat1 centered at the metal binding site. The metal binding site of Nrat1 was characterized by two conserved sequence motifs, i.e., the Asp-Pro-Ser-Asn motif (motif A) and the Ala-Ile-Ile-Thr motif (motif B). Replacement of the Ala-Met-Val-Met motif B of the OsNramp3 manganese (Mn) transporter to that of Nrat1 resulted in a partial gain of Al transport activity and a total loss of Mn in yeast. Conversely, substitution of the motif B of OsNrat1 with that of OsNramp3 altered the Al transport activity. These observations indicated the metal binding site, particularly the motif B, as a key determinant of Al selectivity of Nrat1.

## Introduction

The natural resistance-associated macrophage proteins (Nramps) are widely presented in bacteria, fungi, plants, and mammals (Curie et al., 2000; Nevo and Nelson, 2006). They function as metal ion transporters for a wide range of divalent metal substrates such as Fe^2+^, Mn^2+^, Cd^2+^, Zn^2+^, Co^2+^, Ca^2+^, Cu^2+^, Ni^2+^, and Pb^2+^ (Gunshin et al., 1997). In higher plants, Nramp proteins play major roles in the transport of mineral elements from soil to different organs and tissues of plants. For example, AtNramp1 is found to be localized at the plasma membrane of root cells and functions as a high-affinity transporter for Mn uptake in *Arabidopsis* (Cailliatte et al., 2010). AtNramp3 and AtNramp4 function redundantly to release Fe and Mn from the vacuole (Thomine et al., 2000; Lanquar et al., 2005, 2010). In rice, OsNramp1 transports Fe and Cd in yeast and is suggested to be involved in Cd uptake (Takahashi et al., 2011). OsNramp3 is localized at the plasma membrane of node cells and is involved in distribution of Mn, but not Fe and Cd (Yamaji et al., 2013). The plasma membrane-localized transporter OsNramp5 is the major contributor for Mn and Cd uptake (Sasaki et al., 2012).

Recently, OsNrat1, an Nramp member, was reported to specifically transport Al^3+^ but not divalent metal ions such as Fe^2+^, Mn^2+^, and Cd^2+^, and required for Al tolerance in rice (Xia et al., 2010). In sorghum, SbNrat1, a close homolog of rice OsNrat1, also was shown to selectively transport Al^3+^ (Lu et al., 2017). However, the molecular mechanisms underlying the Al transport selectivity of Nrat1 remain unknown.

Several studies have investigated the relationships between the structure and the function in Nramp proteins. For instance, mutational analysis of the first external loop (Loop I) of NRAMP2/DCT1/DMT1 suggested that Loop I is involved in metal ion binding and specificity (Cohen et al., 2003). The mutation (G185R) in NRAMP2/DCT1/DMT1 not only resulted in a decrease in iron transport but increased the permeability to calcium (Xu et al., 2004). In *Arabidopsis*, three residues (L67, E401, F413) of AtNramp4 have been also shown to play important roles in metal selectivity (Pottier et al., 2015). On the other hand, the crystal structural studies have revealed that Nramp proteins shared a conserved protein fold that was previously found in the amino acid transporter LeuT (Cellier, 2012; Ehrnstorfer et al., 2014). The ScaNramp structure also revealed that a metal binding site consists of conserved aspartate, asparagines, and methionine residues, and a backbone carbonyl from transmembrane segments (TMs) 1 and 6 (Ehrnstorfer et al., 2014). Moreover, the conserved metal-binding site methionine was shown to confer selectivity against the abundant alkaline earth metals calcium and magnesium (Bozzi et al., 2016a). However, the role of the conserved metal-binding site in controlling substrate selectivity is still poorly understood.

In this study, we compared the structure and function of Nrat1 and other initially reported Nramp members in plants by phylogenetic analysis and homology modeling. Furthermore, we performed the site-direct mutagenesis analysis of the conserved metal binding motif in two Nramp proteins, OsNrat1 and OsNramp3, which are known as transporters for Al and Mn (Xia et al., 2010; Yamaji et al., 2013), respectively, and examined their transport activities for Al and Mn. Our results identified a key determinant of Al selectivity of Nrat1, which is essential for Mn selectivity of OsNramp3. It provides novel insights into the molecular basis of Al transport selectivity of Nrat1 and valuable clues to investigate Mn transport selectivity of OsNramp3.

## Materials and Methods

### Sequence and Structure Collection

The amino acid sequences of OsNrat1 homologs from four types of plants, *Oryza sativa, Arabidopsis thaliana, Sorghum bicolor*, and *Zea mays*, were obtained by BLAST (Johnson et al., 2008) using the OsNrat1 sequence as a query in the U.S. National Center for Biotechnology Information (NCBI) reference sequence (RefSeq) database. After eliminating the repetitive sequences, we collected a total of 24 sequences. The structures of prokaryotic Nramp transporters were downloaded from the Protein Data Bank (PDB) database. The 25 Nramp transporters and their NCBI accession numbers are as follows: OsNramp1, XP_015647629; OsNramp2, XP_015632573; OsNramp3, XP_015644306; OsNrat1, XP_015625418; OsNramp5, XP_015645014; OsNramp6, XP_015620405; OsNramp7, XP_015618209; AtNramp1, NP_178198; AtNramp2, NP_175157; AtNramp3, NP_179896; AtNramp4, NP_201534; AtNramp5, NP_193614; AtNramp6, NP_173048; SbNramp1, XP_002459640; SbNramp2, XP_002465667; SbNramp3, XP_002438846; SbNramp4, XP_021317241; SbNramp5, XP_002461772; SbNramp6, XP_002464246; SbNrat1, XP_002451480; ZmNramp1, XP_008670084; ZmNramp4, XP_008670762; ZmNramp5, XP_008652227; ZmNramp6, XP_008665146; ZmNrat1, NP_001334019.

### Sequence Alignment and Phylogenetic Analysis

Multiple-sequence alignment (MSA) was performed by using the T-Coffee server (Di Tommaso et al., 2011). The alignment was produced by combining multiple methods, including mafft_msa, clustalw_msa and t_coffee_msa. Results were subjected to figure production by ESPript version 3.0 (Robert and Gouet, 2014), evolutionary tree building by MEGA6 (Tamura et al., 2013), or evolutionary conservation analysis by ConSurf (Ashkenazy et al., 2010).

Phylogenetic analysis was conducted in MEGA version 6 by the bootstrap neighbor joining method (Saitou and Nei, 1987). Bootstrap method (Felsenstein, 1985) was used for test of phylogeny and the number of bootstrap replications was set to 1000. The evolutionary distances were calculated using the Poisson correction method (Zuckerkandl and Pauling, 1965) and were in the units of the number of amino acid substitutions per site. The analysis involved all 25 amino acid sequences of the Nramp family transporters in the four types of plants. All positions containing gaps and missing data were eliminated. There was a total of 450 positions in the final dataset.

### Evolutionary Conservation Analysis

MSA of the 25 plant Nramp transporters constructed by T-Coffee and the I-TASSER model for the core domain of OsNrat1 (45–502) was used to calculate the position-specific conservation scores by the empirical Bayesian algorithms (Mayrose et al., 2004) in ConSurf (Ashkenazy et al., 2010). The continuous conservation scores are divided into a discrete scale of nine grades for visualization, from the most variable positions (grade 1) colored turquoise, through intermediately conserved positions (grade 5) colored white, to the most conserved positions (grade 9) colored maroon. Scripts for visualizing the protein colored with ConSurf scores were generated and the colored protein was shown in PyMOL (DeLano, 2002).

Normalized conservation scores were also extracted and used to calculate the average conservation score for each structural element and produce figures in GraphPad Prim version 5.

### Vector Construction

The coding region of OsNrat1 and OsNramp3 was amplified from rice (*Oryza sativa*, Nipponbare) root cDNA with high-fidelity PCR (KOD Fx polymerase, Toyobo), and the amplified fragments were cloned into the HindIII/EcoRI, BamHI/EcoRI restriction sites of yeast expression vector pYES2 (Invitrogen), respectively. Site-directed mutagenesis of *OsNrat1* and *OsNramp3* was performed by overlapping PCR (Ho et al., 1989). The wild-type and mutated *OsNrat1* or *OsNramp3* CDS were verified by sequencing. All the PCR primers used are listed in Supplementary Table S1.

### Yeast Assays

The yeast strains used in this study were BY4741 (*MATa his2Δ0 met15Δ0 ura3Δ0*) and *smf1* (*MATa his2Δ0 met15Δ0 ura3Δ0 YOL122c::KanMX4*). Al sensitivity test on agar and complementation of the *smf1* phenotype were performed as described by Xia et al. (2010). For Al sensitivity evaluation, *OsNrat1, OsNramp3, mutated OsNrat1*, or *OsNramp3*, and vector control pYES2 were introduced into yeast strain BY4741 and then spotted on solid media (LPM with 2% galactose for induction of the GAL promoter) containing 0, 200, or 300 μM AlCl_3_ buffered with 5 mM succinic acid. For Al uptake in liquid culture, transformants were selected on uracil-deficient medium and grown in synthetic complete (SC-uracil) yeast solution containing 2% glucose. Cells at mid-exponential phase were harvested and transferred to LPM medium containing 2% galactose. Cells were cultured for 2 h. Then AlCl_3_ was added to the cell culture at the final concentration of 50 μM AlCl_3_. After 6 h incubation with shaking, cells were harvested by centrifugation at 12000 × *g* for 5 min, and washed three times with deionized water (MilliQ; Millipore), dried and then digested with 65% HNO_3_. Al concentration was measured by inductively coupled plasma optical emission spectrometry.

## Results

### Plant Nramp Transporters Fall Into Five Groups on the Phylogenetic Tree

**FIGURE 1:**
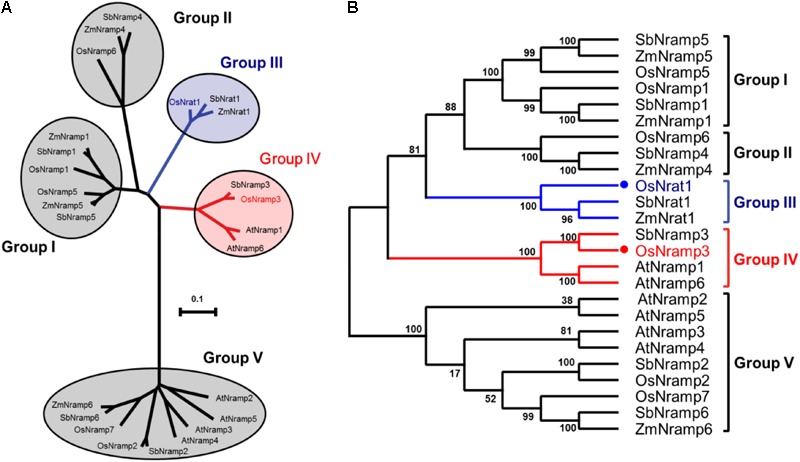
Evolutionary relationships of the plant Nramp family transporters. **(A)** An unrooted phylogenetic tree for the 25 plant metal transporters of the Nramp family was built by MEGA6. The five clades of the evolutionary tree are indicated by circles, with the OsNrat1 containing group (group III) and the OsNramp3 containing group (group IV) colored in blue and red, respectively. The tree is drawn to scale, with branch lengths in the same units as those of the evolutionary distances used to infer the phylogenetic tree. **(B)** Related to **(A)**, the evolutionary tree is shown in a squarish-corner style. The percentages of replicate trees in which the associated taxa clustered together in the bootstrap test (1000 replicates) are shown next to the branches.

The amino acid sequence of OsNrat1 was used to retrieve Nramp homologs in four plant species by BLAST (Johnson et al., 2008). Twenty-four protein sequences were selected for phylogenetic analysis, including six from *Oryza sativa* (OsNrat1, OsNramp1-3, and 5-7), six from *Arabidopsis thaliana* (AtNramp1-6), seven from *Sorghum bicolor* (SbNrat1 and SbNramp1-6), and five from *Zea mays* (ZmNrat1, ZmNramp1, and ZmNramp4-6). The resulting phylogenetic tree includes five main clades corresponding to five distinctive Nramp groups (**Figure 1**). Notably, two known transporters for trivalent Al ion, OsNrat1 (Xia et al., 2010, 2014; Li et al., 2014) and SbNrat1 (Lu et al., 2017), an Nrat1-like transporter of *Zea mays* (ZmNrat1) as well, are located in the same clade (group III) but separated from other divalent ion transporters on the evolutionary tree.

### Nrat1 Transporter Adopts a Conserved LeuT Fold

Sequence alignment of the 25 plant Nramp transporters and the *Staphylococcus Capitis* Divalent Metal Ion Transporter (ScaDMT) reveals that they contain a conserved core domain (amino acid sequence 45–502 in OsNrat1, see Supplementary Figure S1). The available structural information on the Nramp family is limited to the prokaryotic homologues of divalent metal transporters. To understand the mechanism in which trivalent Al is recognized by Nrat1 transporter, we modeled the protein structures of core domain of OsNrat1 and OsNramp3 through an iterative threading algorithm using the I-TASSER server (Roy et al., 2010), as the terminal N- and C- regions with unknown functionality are not as important as the core domain that is highly conserved and constitutes a part of the molecular determinants for ion permeation. To compare the architecture of metal binding sites in the ionic binding state of these two proteins, the Mn-binding structure of ScaDMT (PDBID: 5M95), which showed the highest sequence similarities with OsNrat1 (59%) and OsNramp3 (62%), was used as a template. The estimated TM-score and C-score of OsNrat1 are 0.95 ± 0.05 and 1.68, respectively; while those of OsNramp3 are 0.92 ± 0.06 and 1.50, respectively. Hence, these two models appear to be acceptable.

**FIGURE 2:**
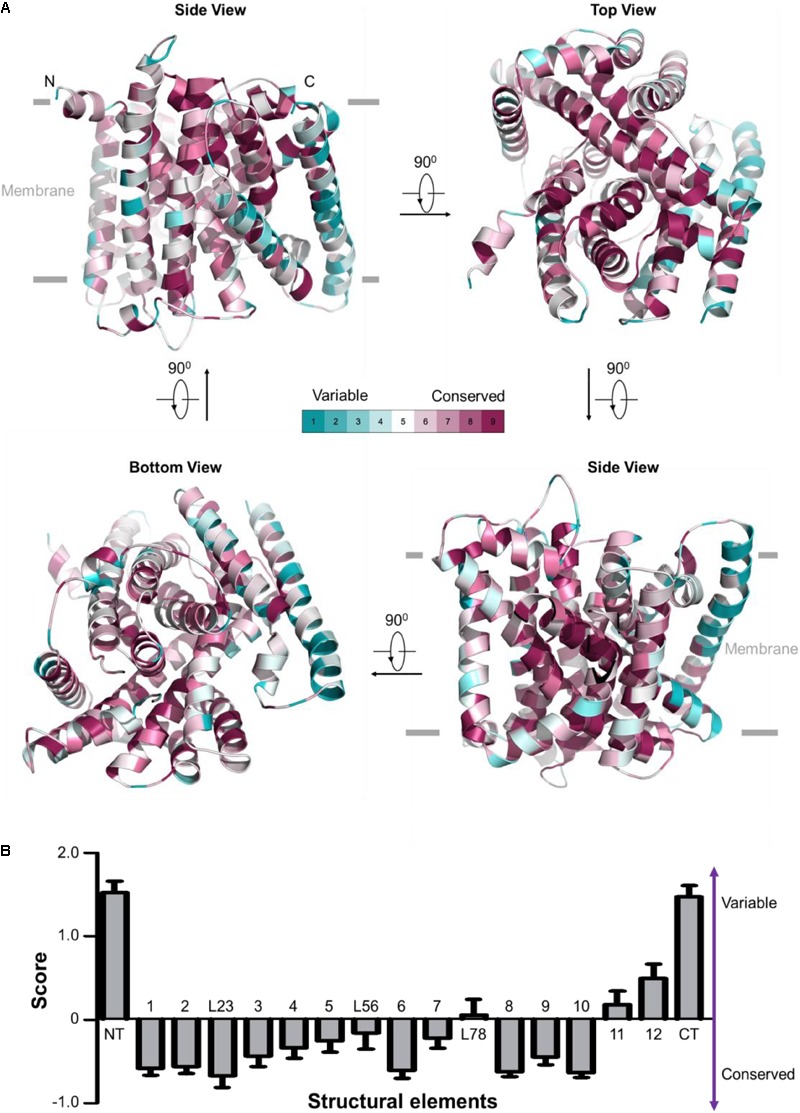
Evolutionary conservation analysis for OsNrat1. The 25 plant Nramp family transporters were used to perform evolutionary conservation analysis by ConSurf. **(A)** Mapping of evolutionary conservation scales for amino acid positions in the core structure of OsNrat1 (45–502). Residues are colored by their conservation grades (1–9) using the color-coding bar, from turquoise to maroon indicating variable to conserved. The structure is shown in four orientations in cartoon representation. **(B)** Normalized evolutionary conservation score for each structural element of OsNrat1. The lowest score indicates that this position is the most conserved in this specific protein calculated using a specific multiple sequence alignment (MSA). Error bars represent SEMs. The NT and CT regions of OsNrat1 are not shown in **(A)**.

OsNrat1 adopts a common LeuT fold (**Figure 2**) that is associated with many prokaryotic Nramp-family transporters, including the *Staphylococcus* divalent metal transporter ScaDMT (59% sequence similarity, PDBID: 5M95), the *Deinococcus radiodurans* Nramp homolog (DraNramp, 56% similarity, PDBID: 5KTE), and the *Eremococcus coleocola* Manganese Transporter (EcoDMT, 56% similarity, PDBID: 5M87). OsNrat1 contains a compact globular domain of 12 transmembrane segments (TMs), of which TMs 1–5 and 6–10 form two inverted repeats of the LeuT fold. Like other LeuT-type transporters, the first TM in each of the two inverted repeats (TM1 and TM6) of OsNrat1 contains two α-helices disrupted by a short discontinuous stretch in the middle (Supplementary Figure S1). Overall, a helical bundle comprising TMs 3–5 and 8–10 forms a semicircular (letter C shaped) structure that wraps partway around a second helical bundle formed by TMs 1, 2, 6, and 7. Substrate transport of the LeuT-type transporters is likely to be coupled with a switch from outward-open to inward-open conformation, through a rigid-body rotation (Shi, 2013) of the moving portion (corresponding to the latter α helical bundle) related to the non-moving portion (corresponding to the former helical bundle).

### The Metal Transport Mechanism of Nrat1 Is Conserved

Evolutionary conservation analysis by the ConSurf server (Ashkenazy et al., 2010) reveals an overwhelming conservation of residues that make up the interior of the Nrat1 cylinder structure (**Figure 2A**), which contains a substrate transport path along the central axis that is perpendicular to the lipid membrane plane. A detailed conservation analysis for each structural element was performed by calculating the normalized evolutionary conservation scores on all amino acid residues. As shown in **Figure 2B**, TMs 1-3, 6, 8-10, along with the L23 loop are highly conserved across all 25 Nramp transporters in plants, suggesting that these conserved elements may play important roles in metal transport. On the contrary, structural elements of NT, CT, TM11 and TM12 in the Nramp family are variable. These observations are consistent with structural and functional analysis of other LeuT-type transporters, indicating a conserved substrate transport mechanism. Similar to other known LeuT-type transporters, the five TMs (TMs 1, 3, 6, 8, and 10) may participate directly in substrate binding and transport. TM2 and TM9, which connect the functional helices TM1/TM3 and TM8/TM10, respectively, may confer transport activity through control of the local conformation. The L23 linker that connects neighboring helices of TM2 and TM3 may parallel the roles of TM2 and TM9.

### The Nrat1 Transporter Contains a Unique Ion Binding Site

**FIGURE 3:**
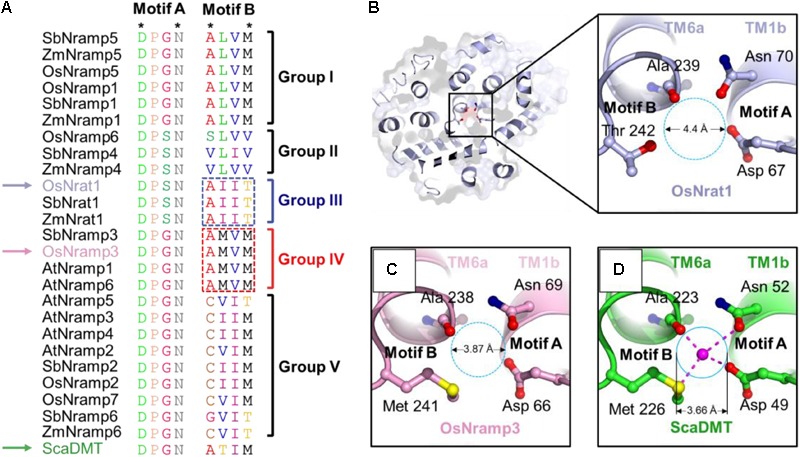
OsNrat1 has a unique metal binding site. **(A)** Sequence alignment of the two signature motifs of the 25 plant Nramp transporters and a bacteria Nramp transporter ScaDMT. The variations of amino acids in the two motifs were marked with different colors. The four residues involved in metal coordination are indicated as stars above the alignment. OsNrat1, OsNramp3, and ScaDMT are indicated by arrows in light blue, pink and green, respectively. The five phylogenetic groups are labeled to the right of the alignment. The amino acids being swapped in Nrat1 and OsNramp3 are marked by dotted rectangles. **(B)** A top view of OsNrat1 from the extracellular side of the membrane **(Left)** with a cartoon representation. The four metal binding residues are shown as colored sticks. The carbon, oxygen, and nitrogen atoms are colored in light blue, red, and tv_blue, respectively. A close-up view of the metal binding site is given in the right panel. **(C)** A close-up view of the metal binding site of OsNramp3 shown with the same orientation as OsNrat1. The coloring code for the atoms are the same as that in **(B)**, except for carbon (pink) and sulfur (yellow). **(D)** A close-up view of the binding site of ScaDMT shown with the same orientation as OsNrat1. The coloring pattern for the atoms is the same as that in **(C)**, except for carbon colored green. The proposed metal binding sites of OsNrat1 and OsNramp3 are indicated by dotted circles, while the metal binding site of ScaDMT is indicated by a solid circle. The approximate diameters of the binding sites are calculated by the equation: *D1* = *D2*^∗^*d1*/*d2. D1* is the diameter of the binding site circle to be calculated. *D2* is the spatial distance between the main-chain oxygen of Ala223 and the side-chain oxygen of Asn52 measured in the crystal structure of ScaDMT by the software PyMOL. *d1* is the diameter of the binding site circle measured in the figure. *d2* is the distance between the main-chain oxygen of Ala223 and the side-chain oxygen of Asn52 of ScaDMT measured in the figure.

The metal recognition site of prokaryotic Nramp transporters is known to be characterized by two structural motifs (**Figure 3** and Supplementary Figure S1), motifs A and B. The highly conserved Asp-Pro-[Gly/Ser]-Asn motif (motif A) occurs in the loop between TM1a and TM1b, as well as the N-terminal portion of TM1b; while the moderate conserved motif (motif B) occurs in the C-terminal portion of TM6a and the loop between TM6a and TM6b. Only four residues, i.e., the first and fourth residues in each motif, are required to coordinate the central metal ion (**Figure 3D**). The Asp and Asn residues in motif A (locating at the first and fourth position, respectively), as well as the fourth residue in motif B, use their side chains to contact the metal ion. By contrast, the first residue in motif B contributes to metal binding by its main-chain carbonyl oxygen. In support of the structural and functional importance of the metal coordination ligands, the Asp and Asn residues in motif A are invariant within the Nramp family and across plant species (**Figure 3A** and Supplementary Figure S1). Notably, the metal ligands in motif B are only moderately conserved (**Figure 3A** and Supplementary Figure S1), suggesting that this motif may contribute to ion-subtype specificity. It is also worth noting that, through refining the proper conformation of terminal residues in each motif, interspace residues of the two motifs may be important for ion binding and selectivity as well.

Sequence alignment results for the two signature motifs show good agreement with the phylogenetic analysis of plant Nramp transporters (**Figure 3A**). Transporters of the Nrat1 group and group II have an Asp-Pro-Ser-Asn sequence pattern of motif A, while other groups consist of a motif A with a uniform Asp-Pro-Gly-Asn sequence. The characteristic sequence patterns of motif B can be clearly divided into five sets, each corresponding to one of the five phylogenetic groups. Among the five sets of motif B, sequences of groups I, III, and IV have the highest conservation. Motif B of group III transporters has an invariant sequence of Ala-Ile-Ile-Thr, while motif B of group I and IV has an identical sequence of Ala-Leu-Val-Met and Ala-Met-Val-Met, respectively. Together, these data indicated that Nrat1 transporters contain a unique pair of sequence motifs which may be critical for mediating metal recognition.

We proceeded to compar the putative metal binding sites from the core domain structural models of OsNrat1 and OsNramp3, with that of the crystal structure of ScaDMT. As shown in **Figures 3B–D**, the architecture of the divalent ion recognition site in OsNramp3 is identical to that observed in ScaDMT. By contrast, the trivalent metal binding site in OsNrat1 appears to be slightly larger than those in OsNramp3 and ScaDMT, as calculated in the two models and the ScaDMT crystal structure (**Figures 3B–D**). These differences are likely caused by a replacement of the Met with a Thr, which contains a shorter side-chain compared to that of a Met, at the fourth residue of motif B.

### The Nrat1 Specific Motif B Is a Key Determinant for Al Transport

To determine the functional importance of the signature motifs, we generated several mutations for OsNrat1 and OsNramp3 (OsNrat1^I240M,I241V,T242M^, OsNrat1^T242M^, OsNramp3^M239I,V 240I,M241T^, OsNramp3^M241T^, see Supplemen-tary Table S2) by exchanging corresponding residues of one protein with another and examined their capabilities on Al and Mn transport as well as OsNrat1 or OsNramp3 as a positive control, respectively.

**FIGURE 4:**
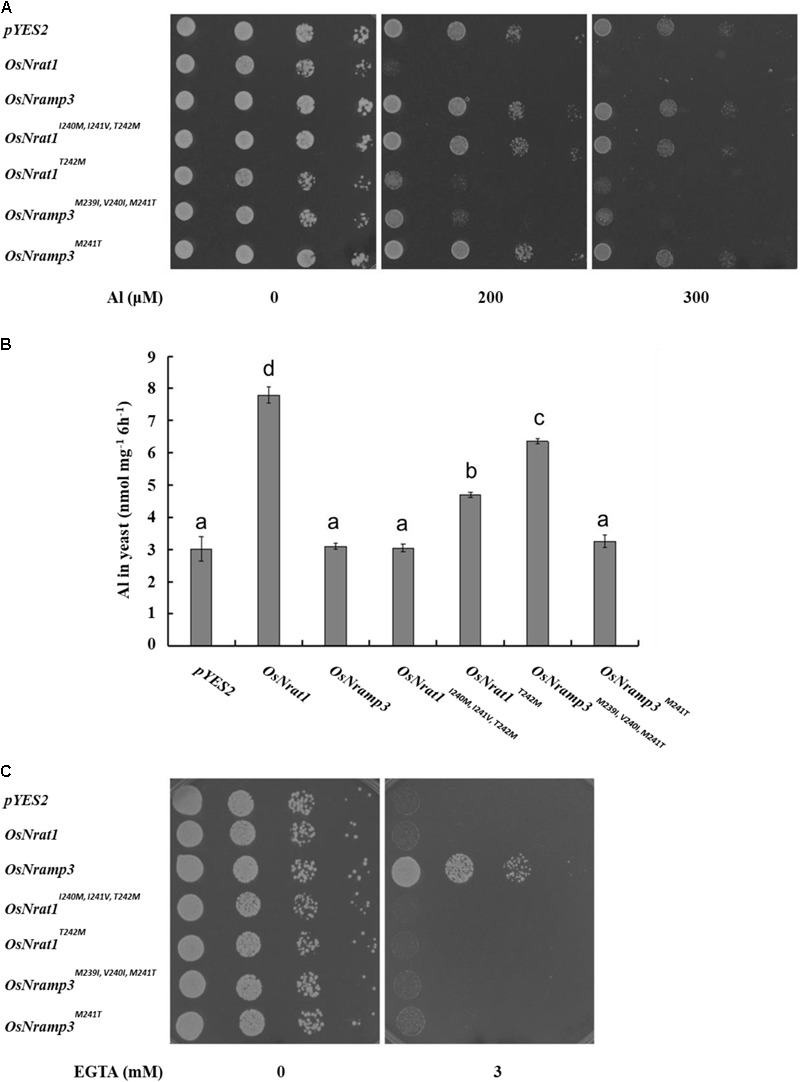
Influence of the signature motif substitution of OsNrat1 or OsNramp3 on transport activity for Al and Mn. **(A)** Effect of mutated OsNrat1 or OsNramp3 on Al tolerance. Yeast strain (BY4741) transformed with empty vector *pYES2, OsNrat1, OsNramp3, OsNrat1^I240M,I241V,T242M^, OsNrat1^T242M^, OsNramp3^M239I,V 240I,M241T^, OsNramp3^M241T^* were spotted on LPM without uracil medium (pH 4.2) buffered with 5 mM succinic acid with or without AlCl_3_ at serial dilutions (from left to right: 10 μl cell suspension with OD 0.2, 0.02, 0.002, and 0.0002) and incubated at 30°C for 3 days. **(B)** Transport activity of mutated OsNrat1 or OsNramp3 for Al. Yeast cells expressing different mutants were exposed to a solution containing 50 μM AlCl_3_ (pH 4.2) for 6 h. Data are mean ± SD of three biological replicates. Different letters above the bars indicate significant differences (P < 0.05, Tukey’s test). **(C)** Complementation of manganese uptake. Transformed *smf1* were grown on a medium (pH 6.0) buffered with 50 mM MES in the presence or absence of EGTA. The plates were incubated at 30°C for 3 days.

In the absence of Al, all the transformants showed similar growth on the plate (**Figure 4A**). However, in the presence of Al, the growth of yeast cells carrying *OsNrat1^T242M^, OsNramp3^M239I,V 240I,M241T^*, or *OsNrat1* was significantly inhibited compared with that of the vector control, while that of *OsNrat1^I240M,I241V,T242M^, OsNramp3^M241T^*, or *OsNramp3* was not (**Figure 4A**). Al uptake also significantly increased in the yeast carrying *OsNrat1^T242M^, OsNramp3^M239I,V 240I,M241T^*, or *OsNrat1* and was not affected in the yeast carrying *OsNrat1^I240M,I241V,T242M^, OsNramp3^M241T^*, or *OsNramp3* (**Figure 4B**). Furthermore, the Al uptake ability of *OsNrat1^T242M^* or *OsNramp3^M239I,V 240I,M241T^* was lower than that of *OsNrat1* (**Figure 4B**). These results suggested that substitution of the intact motif B of OsNrat1 (OsNrat1^I240M,I241V,T242M^) with that of OsNramp3 completely deprived the Al transport activity of OsNrat1, while a single mutation on the fourth residue Thr242Met (OsNrat1^T242M^) resulted in a decrease in Al uptake of OsNrat1, and that replacement of the intact motif B of OsNramp3 (OsNramp3^M239I,V 240I,M241T^) with that of OsNrat1, but not a single mutation on the fourth residue Met241Thr (OsNramp3^M241T^), rendered the Mn specific divalent transporter to gain a function of Al transport.

Subsequently, we performed a complementation test in the *Δsmf1* yeast strain to examine whether mutants of OsNrat1 and OsNramp3 could have transport activity for Mn. As expected, OsNramp3 could restore the growth of a yeast mutant (*smf1*) defective in Mn uptake, while OsNrat1 could not (**Figure 4C**). Surprisingly, as shown in **Figure 4C**, all of the four above mentioned mutations failed to complement the manganese uptake phenotype of the *Δsmf1* mutant yeast. These observations collectively indicate that the Nrat1 specific motif B is both sufficient and required for Al transport, while the OsNramp3 specific motif B, especially the fourth ionic coordination ligand, is only required but not sufficient for Mn transport.

We also studied the functional importance of residues in close vicinity of the signature motifs by sequence exchange between OsNrat1 and OsNramp3 (OsNrat1^A59F, G64A^, OsNrat1^Y 244H^, OsNramp3^F58A, A63G^, OsNramp3^H243Y^, see Supplementary Table S2). As shown in the Supplementary Figure S2, the expression of *OsNrat1^A59F,G64A^, OsNrat1^Y 244H^*, or *OsNrat1* increased the sensitivity of yeast to Al toxicity and the Al uptake in yeast compared with that of the vector control, while that of *OsNramp3^F58A,A63G^, OsNramp3^H243Y^*, or *OsNramp3* did not (Supplementary Figures S2A,B). Furthermore, the Al uptake ability of *OsNrat1^A59F,G64A^* or *OsNrat1^Y 244H^* was lower than that of *OsNrat1* (Supplementary Figure S2B). On the other hand, in contrast to *OsNramp3*, the expression of *OsNrat1^A59F,G64A^, OsNrat1^Y 244H^, OsNramp3^F58A,A63G^*, or *OsNramp3^H243Y^* was not able to complement the growth of the yeast mutant *Δsmf1* under the Mn-limited condition controlling by EGTA (Supplementary Figure S2C). These results indicated that flanking residues of the characteristic motifs are dispensable for Al selectivity but required, at least in part, for Al transport activity. These data also suggested that residues near the metal binding motifs of OsNramp3, but not OsNrat1, are essential for Mn uptake of the transporter.

## Discussion

A number of reported variations in the Nrat1 coding region affect transport activity (Li et al., 2014; Xia et al., 2014; Lu et al., 2017) but not selectivity of Nrat1. We reasoned that careful examination of the metal binding site may facilitate to understand the selectivity of the transporter. The Nramp family of transporters utilizes two separate motifs, each from one of the two discontinuous TMs, to coordinate metal ions (Ehrnstorfer et al., 2014, 2017; Bozzi et al., 2016b). Our bioinformatic and functional analyses demonstrate that the metal binding site, particularly the motif B with a sequence of Ala-Ile-Ile-Thr, is a prominent determinant of Al selectivity for Nrat1.

Motif B of OsNramp3 is probably essential for the selectivity of the transporter. However, the interpretation for selectivity of Nrat1 cannot be directly applied to give a simplified explanation for the selectivity of the Mn specific transporters of the Nramp family, as substitution of mere motif B in OsNrat1 by that of OsNramp3 is not sufficient for the former to gain Mn transport activity. This is consistent with the experimental observations for divalent Nramp transporters reported by Bozzi et al. (2016a). The conserved metal-binding methionine (Met230) of motif B is dispensable in the bacterial DraNramp, as the Met-to-Ala mutant can still enable robust transport of the physiological manganese substrate and similar divalent iron and cobalt. In sharp contrast to the DraNramp, the corresponding Met265Ala mutant of human Nramp2 did not transport any of the tested divalent metals, including Co, Mn, Cd, and Ca. These results indicate a dependency of the functional divergence on sequence and structure context (Bozzi et al., 2016a). Supporting this hypothesis, whereas the single mutation (corresponding to Gln76 of OsNrat1) in TM1b of the mammalian transporter DCT1 (Slc11a2) completely blocked Mn transport, a double mutation (corresponding to Asp74 and Gln76 of OsNrat1) in TM1b restored the activity and altered the metal ion specificity in favor of Fe (Cohen et al., 2003). Moreover, random mutagenesis studies revealed that three residues, Leu67 (in the immediate vicinity of motif A) from TM1a and Glu401/Phe413 from TM10, contributed to the selectivity of AtNramp4 for the uptake of another divalent metal Cd (Pottier et al., 2015).

Our work identified that the Nrat1-type motif B is both sufficient and required for Al transport in Nrat1 and OsNramp3, as one of the key determinants for the Al selectivity. Our results also suggested that the OsNramp3-type motif B is necessary, though not sufficient, for the Mn selectivity of OsNramp3. Identification of the important functions of motif B in substrate selectivity of Nrat1 and OsNramp3 may help further elucidate the selectivity of other Nramp transporters.

## Author Contributions

ZM and JX conceived and designed the experiments. ML, GY, PL, ZW, XZ, XC, SF, and MS performed the experiments. ML, GY, ZM, and JX analyzed the data. ZM and JX wrote the paper.

## Conflict of Interest Statement

The authors declare that the research was conducted in the absence of any commercial or financial relationships that could be construed as a potential conflict of interest.
