# Supplementary material for: Bioinformatic and Functional Analysis of a Key Determinant Underlying the Substrate Selectivity of the Al Transporter, Nrat1
Source: Front Plant Sci. 2018 May 7;9:606. doi: 10.3389/fpls.2018.00606 (PMC5949535; doi:10.3389/fpls.2018.00606)
Supplement: Supplementary file 1 [file Data_Sheet_1.docx]

**SUPPLEMENTARY FIGURE S1** Sequence alignment for 25 plant Nramp transporters and ScaDMT. Secondary structure elements of OsNrat1 from the I-TASSER model are shown below the alignment. Consensus >70 has been indicated for conserved residues. Identical and conserved residues are highlighted by red and yellow grounds, respectively. The two signature motifs involved in metal binding are indicated by black boxes.

**
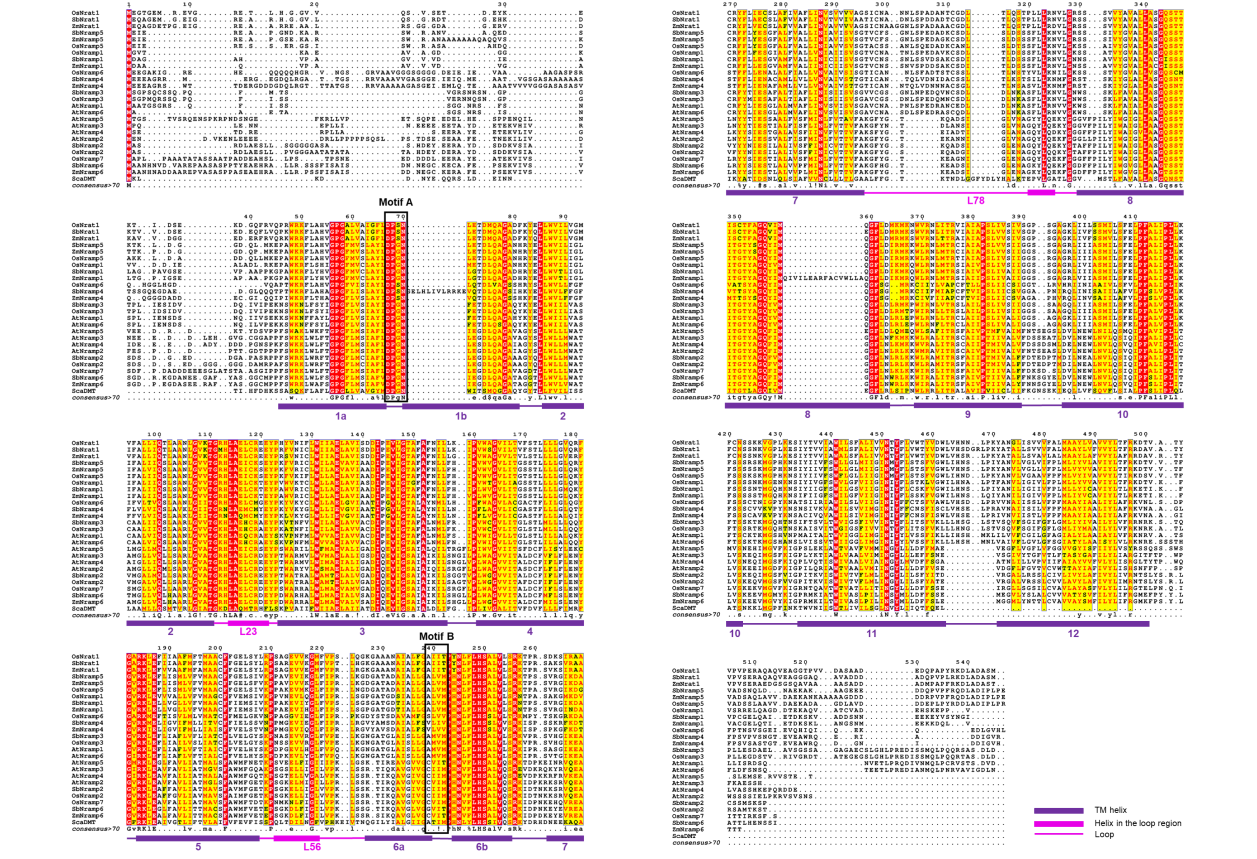
**

**SUPPLEMENTARY FIGURE S2** Influence of the residues’ substitution in close vicinity of the signature motifs of OsNrat1 or OsNramp3 on transport activity for Al and Mn. (**A**) Effect of mutated OsNrat1 or OsNramp3 on Al tolerance. Yeast strain (BY4741) transformed with empty vector *pYES2*, *OsNrat1*, *OsNramp3*, *OsNrat1^A59F, G64A^*, *OsNrat1^Y244H^*, *OsNramp3^F58A, A63G^*, *OsNramp3^H243Y^* were spotted on LPM without uracil medium (pH 4.2) buffered with 5 mM succinic acid with or without AlCl_3_ at serial dilutions (from left to right: 10 μl cell suspension with OD 0.2, 0.02, 0.002, and 0.0002) and incubated at 30 °C for 3 d. (**B**) Transport activity of mutated OsNrat1 or OsNramp3 for Al. Yeast cells expressing different mutants were exposed to a solution containing 50 μM AlCl_3_ (pH 4.2) for 6h. Data are mean±SD of three biological replicates. Different letters above the bars indicate significant differences (P<0.05, Tukey’s test). (C) Complementation of manganese uptake. Transformed smf1 were grown on a medium (pH 6.0) buffered with 50 mM MES in the presence or absence of EGTA. The plates were incubated at 30 °C for 3 d.


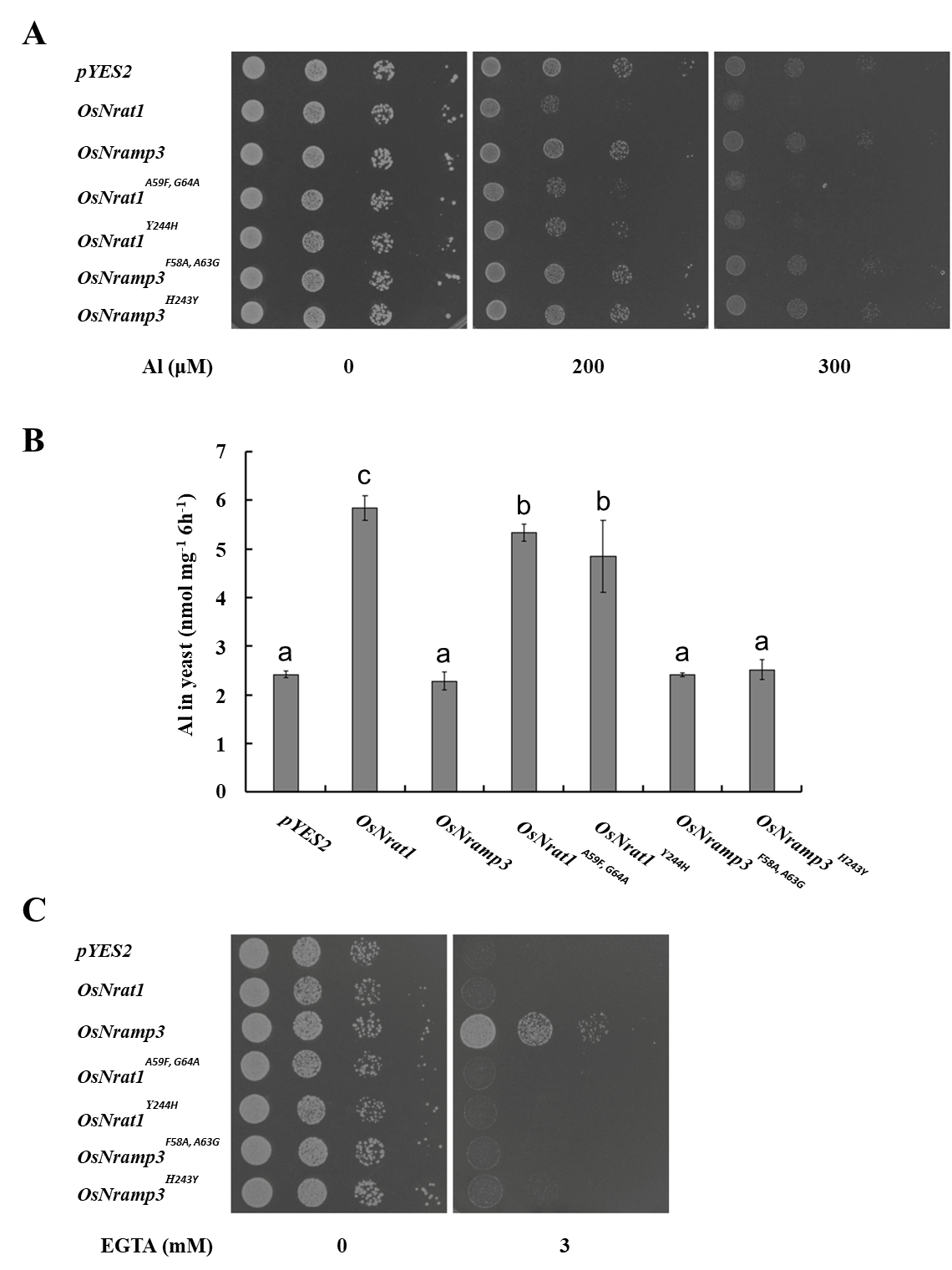


**SUPPLEMENTARY TABLE S1** List of primers used in this study

| Primer | Sequence | Purpose |
| --- | --- | --- |
| ONrYF | 5'- CCCAAGCTTAAAATGGAAGGGACTGGTGAGATG -3'^*^ | Amplification of *OsNrat1* CDS |
| ONrYR | 5'- CCGGAATTCCTACATGGAAGCATCGGCAAGG -3' |  |
| ON3YF | 5'- CGCGGATCCAAAATGAGCGGCCCAATGCAACGC-3' | Amplification of *OsNramp3* CDS |
| ON3YR | 5'- CCGGAATTCCTAATCGAGATCAGAAGCAGTTCG -3' |  |
| M03F | 5'- CTCTTTGGCGCTATGGTGATGCCATACAACTTG -3' | Construction of *OsNrat1^I240M, I241V, T242M^* |
| M03R | 5'- CAAGTTGTATGGCATCACCATAGCGCCAAAGAG -3' |  |
| M04F | 5'- CTCTTTGGCGCTATCATCATGCCATACAACTTG-3' | Construction of *OsNrat1^T242M^* |
| M04R | 5'- CAAGTTGTATGGCATGATGATAGCGCCAAAGAG -3' |  |
| M09F | 5'- GGCCTGGATTTCTGGTGGCCATTGCCTTCCTAGATC -3' | Construction of *OsNrat1^A59F, G64A^* |
| M09R | 5'- GATCTAGGAAGGCAATGGCCACCAGAAATCCAGGCC -3' |  |
| M10F | 5'- CATCACACCACACAACTTGTTC-3' | Construction of *OsNrat1^Y244H^* |
| M10R | 5'- GAACAAGTTGTGTGGTGTGATG -3' |  |
| 3M01F | 5'- CTACTTGGTGCTATCATCACACCGCATAATC-3' | Construction of *OsNramp3^M239I, V240I, M241T^* |
| 3M01R | 5'- GATTATGCGGTGTGATGATAGCACCAAGTAG-3' |  |
| 3M02F | 5'- CTACTTGGTGCTATGGTGACACCGCATAATC-3' | Construction of *OsNramp3^M241T^* |
| 3M02R | 5'- GATTATGCGGTGTCACCATAGCACCAAGTAG-3' |  |
| 3M06F | 5'- GGCCCGGAGCTCTTGTCTCTATTGGGTATATTGATC-3' | Construction of *OsNramp3^F58A, A63G^* |
| 3M06R | 5'- GATCAATATACCCAATAGAGACAAGAGCTCCGGGCC-3' |  |
| 3M07F | 5'- GTGATGCCGTATAATCTTTT-3' | Construction of *OsNramp3^H243Y^* |
| 3M07R | 5'- AAAAGATTATACGGCATCAC -3' |  |

* The adding restriction sites were underlined.

**SUPPLEMENTARY TABLE S2** Summary of mutants analyzed in this study

| Mutant | Mutation sites |
| --- | --- |
| OsNrat1^I240M, I241V, T242M^ | I240, I241 and T242 in OsNrat1 were replaced with the corresponding residues M239, V240 and M241 in OsNramp3 |
| OsNrat1^T242M^ | T242 in OsNrat1 was replaced with the corresponding residues M241 in OsNramp3 |
| OsNrat1^A59F, G64A^ | A59 and G64 in OsNrat1 were replaced with the corresponding residues F58 and A63 in OsNramp3 |
| OsNrat1^Y244H^ | Y244 in OsNrat1was replaced with the corresponding residues H243 in OsNramp3 |
| OsNramp3^M239I, V240I, M241T^ | M239, V240 and M241 in OsNramp3 were replaced with the corresponding residues I240, I241 and T242 in OsNrat1 |
| OsNramp3^M241T^ | M241 in OsNramp3 was replaced with the corresponding residues T242 in OsNrat1 |
| OsNramp3^F58A, A63G^ | F58 and A63 in OsNramp3 were replaced with the corresponding residues A59 and G64 in OsNrat1 |
| OsNramp3^H243Y^ | H243 in OsNramp3 was replaced with the corresponding residues Y244 in OsNrat1 |
